# Nutrients in Fish and Possible Associations with Cardiovascular Disease Risk Factors in Metabolic Syndrome

**DOI:** 10.3390/nu10070952

**Published:** 2018-07-23

**Authors:** Christine Tørris, Milada Cvancarova Småstuen, Marianne Molin

**Affiliations:** 1 Faculty of Health Sciences, Oslo Metropolitan University, NO-0130 Oslo, Norway; Milada-Cvancarova.Smastuen@oslomet.no (M.C.S.); Marianne.Molin@oslomet.no (M.M.); 2 Department of Health Sciences, Bjorknes University College, NO-0456 Oslo, Norway

**Keywords:** fish consumption, lean fish, fatty fish, metabolic syndrome, cardiovascular risk, cardiovascular disease, diet, protein, amino acids, nutrients

## Abstract

Non-communicable diseases (NSDs) are responsible for two-thirds of all deaths globally, whereas cardiovascular disease (CVD) alone counts for nearly half of them. To reduce the impact of CVD, targeting modifiable risk factors comprised in metabolic syndrome (e.g., waist circumference, lipid profile, blood pressure, and blood glucose) is of great importance. Beneficial effects of fish consumption on CVD has been revealed over the past decades, and some studies suggest that fish consumption may have a protective role in preventing metabolic syndrome. Fish contains a variety of nutrients that may contribute to health benefits. This review examines current recommendations for fish intake as a source of various nutrients (proteins, *n*-3 fatty acids, vitamin D, iodine, selenium, and taurine), and their effects on metabolic syndrome and the CVD risk factors. Fatty fish is recommended due to its high levels of *n*-3 fatty acids, however lean fish also contains nutrients that may be beneficial in the prevention of CVD.

## 1. Introduction

Cardiovascular disease (CVD) is the most common cause of death worldwide [1], being responsible for more than 40% of the deaths from non-communicable diseases (NCDs) [2]. CVD includes coronary heart diseases (CHD), such as angina and heart attack, cerebrovascular disease (stroke) and peripheral arterial diseases. The underlying disease process of CVD is atherosclerosis, a complex pathological process in the walls of blood vessels.

Several risk factors promote the process of atherosclerosis, and both behavioral (e.g., tobacco, physical activity, diet, and alcohol) and metabolic risk factors (e.g., obesity, raised blood lipids, blood pressure, and blood sugar) play a key role in its etiology [1]. Some of these metabolic risk factors were described by Kylin as early as in the 1920s, as the clustering of hypertension, hyperglycemia, and gout [3]. Today, these metabolic risk factors are known as metabolic syndrome (MetS), and a clinical definition was agreed upon through a Joint Interim Statement (JIS) in 2009, after several attempts to unify the criteria for MetS [4]. In the JIS definition, a presence of any three of the five given risk factors (abdominal obesity, elevated triglyceride (TG), reduced high-density lipoprotein cholesterol (HDL-C), elevated blood pressure (BP), and elevated fasting glucose) constitutes a diagnosis of MetS [4] (Table 1).

Abdominal obesity appears to precede the appearance of the other MetS components [5], where an expansion of adipose tissue due to adipocyte hypertrophy leads to an inflammatory response in the fat tissue due to the infiltration of macrophages and other immune cells that release pro-inflammatory cytokines, such as tumor necrosis factor alpha (TNF-α) and interleukin 6 (IL-6) [3,6]. TNF-α influence the immune system by increasing the permeability and adhesiveness of the blood vessels, while IL-6 stimulates hepatocytes to synthesize C-reactive protein (CRP) [7], which is a systemic inflammation marker.

Fish is recommended as a part of a healthy diet [8] and it is considered to be a key component of a cardio-protective diet [9]. Furthermore, fish is an important source of various nutrients, such as protein, *n*-3 fatty acids, vitamin D, iodine, and selenium [10], which may contribute to a healthier metabolic profile [11,12].

A recent meta-analysis from Iran found an inverse association between fish consumption and risk of all-cause and total cardiovascular mortality [13]. However, regional differences were revealed for this association, whereas higher fish intake was associated with higher risk of all-cause and cardiovascular mortality in Western countries, but not in Asians [13]. These findings may suggest that types of fish/species consumed, methods of fish preparation, and potential local contaminants should be considered in addition to other possible confounders, such as regular food items consumed together with the fish.

Consumption of fatty fish has been suggested to reduce the risk of CVD, which is mainly due to its high level of *n*-3 fatty acids [14]. Beneficial effects of fish consumption (in general) on CVD have been revealed over the past decades [14,15,16], and several studies have suggested an inverse relationship between fish consumption and heart failure [17], cerebrovascular disease [18], coronary calcification [19], ischemic stroke [20], and sudden coronary death risk [21]. Other studies suggest that fish consumption prevent, or improve metabolic health and thus has a role in MetS prevention [22].

Targeting the modifiable risk factors of MetS is of great importance for public health. This review aims to examine current recommendations for fish intake as a source of various nutrients (proteins, *n*-3 fatty acids, vitamin D, iodine, selenium, and taurine) and their possible effects on MetS and the CVD risk factors. To explore how fish intake affects CVD risk, we systematically reviewed prospective and intervention studies investigating the effects of fish consumption on the CVD risk factors of the MetS.

## 2. Method

Literature search was performed in PubMed to identify studies examining the associations between fish consumption and the possible effects on MetS and the CVD risk factors of the MetS. Combined search terms were: (1) fish consumption and metabolic syndrome; (2) fish consumption and each of the individual risk factors comprising MetS (e.g., waist circumference, lipid profile, blood pressure, and glucose); and (3) fish consumption and cardiovascular risk.

**Figure 1:**
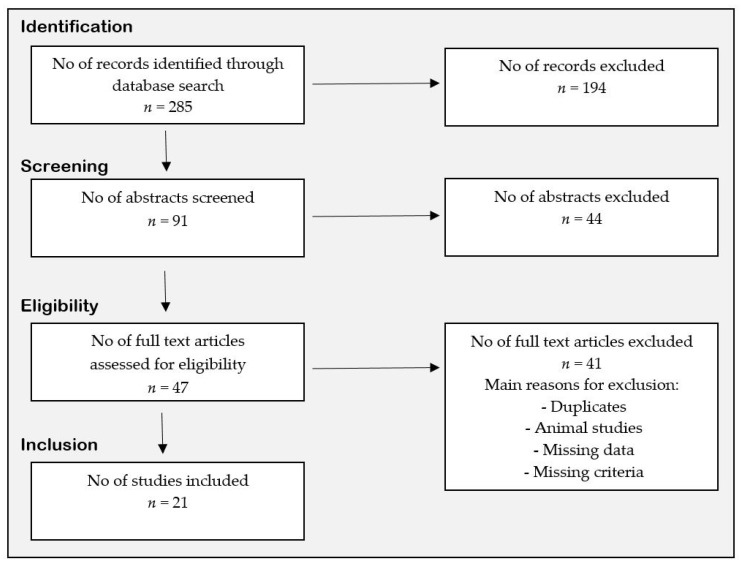
Flow diagram of the review process.

The search was restricted to papers that are written in English, and animal studies were excluded. The selection process is illustrated via a flow diagram (Figure 1). All intervention, prospective cohort, and cross-sectional studies (conducted among adults) investigating the association between fish consumption and MetS were included, however, only intervention and prospective cohort studies investigating the effect of fish consumption on the CVD risk factors were included. Potential abstracts and full-text articles were screened and assessed for eligibility, and the included studies are presented (Table 2, Table 3 and Table 4). Abstracts, letters, or reviews were not included, but they were inspected for additional references that met the inclusion criteria. In addition, the reference lists of the included studies and relevant published reviews were examined to identify additional papers for possible inclusion. The review procedure was performed in accordance with the PRISMA statement for review reporting [23], and a protocol of the study selection was made. The last search was done on 8 December 2017.

## 3. Results

The literature search identified 285 citations, of which 108 were found in the first search (fish consumption and metabolic syndrome). In the second search (fish consumption and the individual risk factors comprised MetS), 120 studies were identified (e.g., waist circumference: 6, lipid profile: 28, blood pressure: 46, and glucose: 40). In the third search (fish consumption and cardiovascular risk), 57 studies were identified. Finally, 11 studies examining the associations between fish consumption and MetS were included (Table 2), and 15 studies (intervention and follow-up) examining the associations between fish consumption and the CVD risk factors comprised MetS were included (Table 3 and Table 4).

### 3.1. Fish Consumption and Metabolic Syndrome

The included studies are one intervention study, two follow-up studies, and eight cross-sectional studies. The intervention study [24] (8-weeks, energy restricted (−30%): 150 g cod 3 times/week, 150 g cod5 times/week, or no seafood) found no association between fish consumption and MetS, while an inverse association between fish consumption and MetS was identified in both of the follow-up studies [25,26]. In these two observational studies, the participants did not have MetS at the beginning of the study. Regarding the cross-sectional studies, six out of eight studies found an association between fish consumption and MetS [27,28,29,30,31,32]. However, for two of these [30,31], the association was found only for lean fish and men (Table 2). Few studies have investigated the possible differences between lean and fatty fish consumption and MetS. A recent meta-analysis found a significant inverse association between total fish consumption and the risk of MetS when pooling data from prospective cohort studies, however, no significant association was found after pooling the cross-sectional studies [33].

### 3.2. Fish Consumption and the CVD Risk Factors of Metabolic Syndrome

The included studies comprised in 13 intervention studies (Table 3), and two follow-up studies (observational) (Table 4). All studies identified associations between fish consumption and CVD risk factors of MetS, however, none of the studies found association between fish consumption and blood glucose.

Regarding waist circumference (WC), four of the intervention studies identified the associations between fish consumption and reduced WC, whereas three identified associations for lean fish [24,46,47] and one for fatty fish [46]. The greatest decrease in WC was found in overweight and obese adults, where a 3.4 cm reduction relative to the control group was found among those in the lean fish consuming group (cod 5 t/week) [24]. However, all of the participants in this study followed an energy restricted diet (−30% from calculated total energy expenditure). Recently, a large Norwegian study (*n* = 23,907), found that lean fish consumption was associated with a 1.15 cm decrease in WC among men, while the consumption of fatty fish was associated with an increased WC for both genders (women: 0.97 cm, men: 0.60 cm [48]. In contrast to this study, a European cohort study found no association between the consumption of lean fish and WC, but identified an annual decrease in WC with a −0.01 cm per 10 g higher fatty fish consumption daily [49].

Several, but not all, of the intervention studies revealed a decreased TG and an increased HDL associated with consumption of fatty, as well as lean fish. A decreased TG and an increased HDL was also reported in one of the follow-up studies, and here particularly lean fish was associated with a reduced TG and a healthier lipid profile [48].

For most of the studies, fish consumption decreased BP, but this finding was not consistent. In the intervention studies, lean fish consumption was associated with decreased BP, both in cardiac patients randomized to lean fish, fatty fish, or lean meat (control) [38], and in patients with MetS randomized to lean fish or no fish/seafood [47]. On the other hand, one study found an increased BP after lean fish consumption [24]. However, fatty fish consumption decreased BP [37,41]. Lean fish consumption was additionally associated with lower BP in one follow-up study [48]. Also, a previous European cross-sectional study among elderly participants (aged 65 to 100 years) found reduced BP among those with a high intake of fish (>300 g/week), however only for SBP [15].

Fish consumption and possible associations with blood glucose have previously been investigated in cross-sectional studies, and both a reduction in fasting blood glucose [15] and a slightly higher non-fasting blood glucose level have been found among those with high fish consumption, as compared to those with a low intake of fish [31]. Still, improved glucose metabolism has been found in obese participants receiving a healthy diet containing fish [50,51].

## 4. Nutritional Contribution of Fish

Fish is an important source of a variety of nutrients, such as *n*-3 fatty acids, proteins, selenium, iodine, vitamin D, and taurine [10] (Table 5). The current dietary recommendations from various governing bodies recommend the consumption of fish one to three times a week (Table 6). Fish can be classified as lean, medium-fatty, or fatty depending on the amount of fat in its body tissue, where fatty fish contains more than 8 g of fat per 100 g, medium-fatty fish contains 2–8 g of fat per 100 g, and lean fish contains less than 2 g of fat per 100 g [10]. While the amount of fat varies considerably in fatty fish, it is relatively stable in lean fish. The content and concentration of nutrients vary between the species, and the largest differences are between fatty and lean fish. Fatty fish has a higher level of *n*-3 fatty acids and the fat-soluble vitamin D, but in contrast lean fish contains more iodine and taurine [10].

### 4.1. Proteins and Amino Acids

Fish contains a unique combination of high-quality proteins. Both a greater satiety level and a slower decline in satiety have been observed among participants consuming fish (Mustelus antarcticus) when compared to participants consuming beef or chicken [60]. In contrast, a reduction in energy intake after a meal containing fish protein (cod) without a reduction in satiety has also been observed (2765 vs. 3080 KJ), with no later energy compensation after the meal [61]. Further, fish (tuna) has been found to be more effective than turkey and egg in reducing both appetite and food intake, and stimulating a greater insulin response as compared to turkey and egg [62].

Fish proteins have been found to have beneficial effects on lipid profiles. A Norwegian randomized controlled trial (RCT) reported a reduction in both fasting and postprandial circulating TG concentrations in participants consuming proteins from lean fish (e.g., cod, pollock, saithe, and scallops), when compared to the intake of non-seafood protein sources (lean meat: chicken, beef, turkey, pork, egg, and low-fat milk) [36]. Beneficial changes in lipid profiles have also been observed in animal studies, both in rabbits receiving fish protein (cod) when compared to casein or milk protein [63] and in rats receiving fish protein as compared to casein or soy protein [64,65,66]. In animal studies, dietary proteins have been suggested to regulate lipid metabolism and to slow both the absorption and the synthesis of lipids and to further promote lipid excretion [67]. The responsible mechanisms of fish protein on lipid metabolism has not been fully identified, however, both amino acid composition and bioactive peptides may play a role [67]. Furthermore, dietary proteins from cod have been found to improve insulin sensitivity in insulin-resistant individuals, when compared to those consuming other animal proteins [68].

Beneficial effects of taurine on cardiovascular risk factors have been proposed [69,70,71,72], and both a reduction in body weight [73], beneficial effects on blood lipids [73,74], anti-atherosclerotic, and anti-inflammatory effects have been observed [71]. The anti-obesity effects of taurine may partly be due to suppression of inflammation in adipose tissue [75]. Furthermore, taurine supplementation has been found to increase adiponectin levels, and decrease markers of inflammation (high-sensitivity C-reactive protein) [76]. Recently, taurine has been found to lower BP, both in humans where the participants received either taurine supplementation (1.6 g/day) or a placebo for 12 weeks. Here, the mean SBP reduction for taurine/placebo was 7.2/2.6 mm Hg, and DBP was 4.7/1.3 mm Hg [77]. Such associations have also been found in rats [78]. This lowering effect may be due to the improved vascular function of taurine, possibly through antagonism of Angiotensin II action influencing the blood pressure [79]. Furthermore, taurine may affect the regulation of blood glucose, and it has a role in beta-cell function attenuating cell injury as induced by stress in the islets [80].

### 4.2. n-3 Fatty Acids

*n*-3 fatty acids are polyunsaturated fatty acids (PUFA) that are naturally found in plants (e.g., soybeans, mustard, walnut, linseed) and seafood, such as fish and algae. The marine long-chain *n*-3 fatty acids eicosapentaenoic acid (EPA) and docosahexaenoic acid (DHA) are mainly present in fatty fish [81], however, lean fish is also a source to *n*-3 fatty acids as it contains approximately 260 mg *n*-3 per 100 g (Table 4). EPA and DHA are responsible for biological actions, such as maintaining the cell membrane, modulating inflammatory processes, and decreases the secretion of pro-inflammatory cytokines [82] affect both lipid metabolism and thrombosis [83]. Furthermore, *n*-3 fatty acids have been shown to regulate pathways controlling fat storage and fat mobilization and to decrease lipid accumulation processes [84].

An intake of two to three portions/week of a variety of seafood provide a recommended intake of EPA and DHA (250.0 mg/d) [55], however not all individuals fulfil the recommended intake [81].

Several decades ago, Bang and Dyerberg discovered the cardio-protective effects of *n*-3 fatty acids when they found that both the Greenland Eskimos food and blood contained a very high level of *n*-3 fatty acids, when compared with a Danish cohort with higher frequency of ischemic heart disease compared to the Eskimos [85]. Today, current evidence supports the beneficially effects of *n*-fatty acids on cardiovascular risk factors [86], where beneficial effects of *n*-3 fatty acids (fish oil) on WC [87], decreased TG [87,88], increased HDL-C [88,89], and reduced blood pressure [44,87] have been demonstrated. However, a recent meta-analysis of almost 80,000 patients (coronary heart disease, stroke, or diabetes) did not find any reduced risk of cardiovascular outcomes after intake of *n*-3 fatty acid supplements, indicating that the cardioprotective effects of *n*-3-fatty acids for these patients may have been overestimated. Whether or not fish oil supplements are beneficial for primary prevention of heart disease for healthy people, remains presently uncertain [90].

Beneficial effects of *n*-3 fatty acids on MetS and CVD have been proposed [91]. *n*-3 fatty acids have also been associated with reduced risk of MetS in follow-up studies [25,26], however not all found such association [34]. Potential anti-inflammatory effects of EPA and DHA have been revealed (in vitro studies), where *n*-3 PUFAs have been found to modulate pro-inflammatory cytokines, such as TNF-α and IL-6 in macrophages [92], and DHA, in particular, seems to decrease TNF-α levels [93]. Nevertheless, even though fish oil supplements are popular [94], *n*-3 fatty acids may be more efficacious when consumed in fish rather than equivalent amounts provided as fish oil capsules. A lower *n*-3 intake from fish resulted in higher increments in plasma EPA and DHA concentration when compared to capsules, where a two- to nine-fold higher dose of EPA and DHA from capsules was needed to give the same increments as fish [95]. *n*-3 FAs from fish and sea mammals have been associated with lower blood pressure, TG, 2-hour glucose and higher HDL-C, when high intake of *n*-3 FAs was compared to low intake of *n*-3 FAs [96].

### 4.3. Vitamin D

Vitamin D is a fat-soluble vitamin that is important for bone health and calcium homeostasis, and it may also have an anti-inflammatory effect on human immune cells [97]. Vitamin D is naturally found in fish, and it is also derived in the skin by the effect of ultraviolet rays from sunlight. Fatty fish is a major food source of vitamin D [98], however, the vitamin D content in farmed salmon seems to be declining, which is possibly due to changes in fish feeds over the years [99]. However, recommended levels of fish consumption are usually not enough to optimize vitamin D status alone, and the fortification of different foods, such as margarine, is common in many countries [100].

The recommended intake of vitamin D in Europe is estimated to 15 microgram/day, in order to achieve a serum 25(OH)D concentration near or above the target of 50 nmol/L [59]. However, not all individuals fulfil the recommended intake and high prevalence of vitamin D deficiency has been observed both in Europe [101,102] and in the United States [103].

Recently, low vitamin D level (<30 ng/mL) was associated with increased risk of MetS (OR 1.90, 95% CI 1.26 to 2.85), and several of the cardiovascular risk factors comprised in MetS; high TG levels (OR 1.55, 95% CI 1.13 to 2.35), and low HDL-C levels (OR 1.60, 95% CI 1.19 to 2.40) [104]. This have also been found in other studies; MetS [105], WC [106,107], HDL-C [107,108,109], BP [107], and glucose [110].

### 4.4. Iodine and Selenium

Both iodine and selenium are trace elements that are required for the thyroid function [111]. While iodine is important for the normal functioning of the thyroid gland [10], through the production of the hormones thyroxine (T4) and triiodinethyroxine (T3), the primary functions of selenium is to be a co-factor in antioxidant activities and thyroid hormone metabolism [112]. Diseases in the thyroid gland influence body weight, thermogenesis, and lipolysis in adipose tissue, thus hypothyroidism is often related to weight gain, decreased thermogenesis, and metabolic rate [113].

The highest levels of iodine are found in lean fish, where ten times more iodine is found in cod and haddock when compared to fatty fish, such as salmon and trout [52]. Among adults, the recommended daily intake (RDI) of iodine is 150 microgram/day, which is considered to be the appropriate amount to allow for normal T4 production without stressing the thyroid [112]. Several studies have reported of low intake of iodine and iodine deficiency [114,115,116,117,118]. On the other hand, Iceland has been known for its population’s good iodine status, which is possibly due to their high intake of lean fish and here the most commonly consumed fish is haddock and cod [115,119].

Previously, higher thyroid volume has been found among patients with MetS, as compared to healthy controls without MetS [120]. Obesity may be an independent risk factor to iodine deficiency, and lower urinary iodine has been found in obese women, in comparison with healthy non-obese women [121]. Furthermore, low intake of iodine and no intake of iodine supplements have been associated with obesity in Denmark [117].

For selenium, the recommended daily intake (RDI) is 50 microgram/day for women, and 60 microgram/day for men [58]. Selenium intake and status have been reviewed, and found to be suboptimal both in Europe and in the Middle Eastern countries [122]. One exception is Finland where the selenium levels are now sufficient after a nationwide selenium fertilization programme initiated due to this country having the lowest levels in the world in the early 1980s [123]. In a Spanish population (*n* = 2009, 50% men), 25% did not meet the recommended intake [124], and as much as half of a group of postmenopausal women (*n* = 97) in New Zealand did not meet the recommended intake of 50 microgram/day [114].

A reverse association between blood selenium levels and blood pressure has been reported in men, but not in women [125]. Possible harmful effects of mercury on blood pressure may be attenuated by high levels of selenium [126].

## 5. Conclusions

This review has examined the state of knowledge on the current known beneficial nutrients in fish (*n*-3 fatty acids, proteins, selenium, iodine, vitamin D, and taurine), and their possible associations with the CVD risk factors comprising MetS.

In the recommendations, dietary advice emphasize intake of fatty fish due to its high levels of *n*-3 fatty acids. However, lean fish contains numerous nutrients that may be beneficial in the prevention of CVD, indicating that also lean fish should be included in the diet when targeting these modifiable risk factors that are comprised in MetS.

## Figures and Tables

**Table 1:** Criteria for diagnosing the metabolic syndrome (MetS) [4].

Measure	Cut Points
Waist circumference *	Men: ≥94 cm
	Women: ≥80 cm
serum-HDL cholesterol	Men: <1.0 mmol/L (40 mg/dL)
	Women: <1.3 mmol/L (50 mg/dL)
serum-triglyceride	>1.7 mmol/L (150 mg/dL)
Systolic blood pressure	≥130 mm Hg
Diastolic blood pressure	≥85 mm Hg
Fasting serum-glucose	≥5.6 mmol/L (100 mg/dL)

* Population- and country-specific definitions: Caucasian, Middle East, Mediterranean, Sub-Saharan African, Ethnic Central and South American. HDL: High-density lipoprotein cholesterol.

**Table 2:** Studies on fish consumption and metabolic syndrome (MetS).

Reference	Design	Results
Ramel et al., 2009 [24]	RCT	No association (8-week intervention, energy restricted * (−30%): 150 g cod 3 times/week, 5 times/week, or no seafood as control)
Baik et al., 2010 [25]	3-years follow-up	Men: Reduced risk of MetS with average daily fish consumption (40–70 g), when compared with less than once a week, OR 0.43 (95% CI 0.23 to 0.83). Women: No associations
Kim et al., 2016 [26]	25-years follow-up	Men and women: Non-fried fish consumption inversely associated with incidence of MetS, age adjusted HR 0.51 (95% CI 0.40 to 0.64) from the highest quintile (≥5 week)
Karlsson et al., 2017 [27]	Cross-sectional	Men and women: Total fish intake was inversely associated with MetS, adjusted OR 0.75 (95% CI 0.57 to 0.97).
Kouki et al., 2011 [28]	Cross-sectional	Men: Fish consumption (10 g/day) associated with reduced risk of having MetS, OR 0.97 (95% CI 0.94 to 1.00) Women: No associations
Lai et al., 2013 [34]	Cross-sectional	No association
Pasalic et al 2011 [35]	Cross-sectional	No association
Ruidavets et al., 2007 [29]	Cross-sectional	Men: Inverse association between fish consumption and prevalence of MetS, when comparing highest tertile to lowest, OR 0.57 (95% CI 0.38 to 0.86).
Tørris et al., 2016 [31]	Cross-sectional	Men and women: Fish consumption was associated with lower risk of MetS in the age group 60–70 years (OR 0.64, 95% CI 0.45 to 0.91), compared to the younger age groups. However, only lean fish consumption was associated with a reduced risk of having MetS, no association for fatty fish consumption.
Tørris et al, 2016 [30]	Cross-sectional	Men and women: Fish consumption once a week or more was associated with lower risk of MetS, OR 0.83 (95% CI 0.74 to 0.93), compared to fish consumption less than once a week. Only lean fish was associated with lower risk of having metabolic syndrome, no association for fatty fish consumption.
Zaribaf et al., 2014 [32]	Cross-sectional	Women in the highest tertile of fish consumption were less likely to have MetS, compared to those in the lowest tertile, OR 0.35 (95% CI 0.14 to 0.88).

* −30% from calculated total energy expenditure, approximately 600 kcal/day. MetS: Metabolic syndrome.

**Table 3:** Intervention studies on fish consumption and the metabolic syndrome risk factors.

Reference/Country	Participants	Intervention	Results
Aadland et al., 2015 [36] Norway	20 healthy adults (7 men, 13 women)	4-week, crossover: 1: 60% of proteins lean-seafood 2: No seafood (lean meat and 3.3 g cod liver oil)	Lean fish reduced TG Change −0.17 ± 0.06 mmol/L HDL no association
Bao et al., 1998 [37] Australia	69 overweight nonsmoking men and postmenopausal women, using hypertensive	16-week, parallel: 1: Fatty fish (3.65 g *n*-3 FA) & energy restricted (−2MJ/d) 2: Energy restricted (−2MJ/d) 3: Fatty fish (3.65 g *n*-3 FA) 4: Control	Fatty fish reduced BP SBP −6.0 ± 1.4 mm Hg DBP −3.0 ± 1.4 mm Hg
Erkkila et al., 2008 [38] Finland	33 adults (27 men, 6 women) with coronary heart disease	8-week, parallel: 1: Fatty fish (4 meals/week) 2: Lean fish (4 meals/week) 3: Control (lean meat)	Lean fish reduced BP SBP −3.5 ± 3.2 mmHg DBP −4.6 ± F 3.6 mmHg
Hagen et al. 2016 [39] Norway	38 healthy adults	4-week, parallel 1: Lean fish (750 g/w) 2: Fatty fish 3: Control (lean meat)	High intake of fatty fish, but not lean fish reduced TG and increased HDL
Hallund et al., 2010 [40] Denmark	68 healthy men	8-week, parallel: 1: Trout (marine diet) 2: Trout (vegetable diet) 3: Control (chicken)	No association (BP, TG, HDL, glucose)
Lara et al., 2007 [41] UK	48 healthy adults (16 men)	4 week: Salmon (4-weeks) followed by no-fish (4-weeks)	Fatty fish reduced TG −0.13 (−0.24 to −0.1) Fatty fish increased HDL 0.09 (0.01 to 0.15) Fatty fish decreased BP SBP −4.6 (−7.1 to −2.0) mmHg DBP −2.9 (−5.0, −0.9) mmHg
Lindquist et al., 2009 [42] Sweden	35 overweight men	6-week, cross-over 1: Herring 5 day/week 2: Chicken and pork	Fatty fish decreased TG −0.35 mmol/l Fatty fish increased HDL +0.05 mmol/l BP no association
Lindquist et al., 2007 [43] Sweden	15 healthy obese adults	4-week, cross-over 1: Herring 5 day/week 2: Chicken and pork	Fatty fish increased HDL +0.09 mmol/l TG no association
Ramel et al., 2010 [44] Iceland, Spain, Ireland	324 overweight/obese healthy adults	8-week, parallel Energy restricted * (−30%): 1: Lean fish (cod) 3 times/week 2: Fatty fish (salmon) 3 × 150 g/week3: Fish oil (DHA/EPA) 4: Control (No seafood)	Fatty fish decreased DBP compared with lean fish, no association with control group
Ramel et al., 2009 [24] Iceland	126 overweight/obese healthy adults	8-week, parallel Energy restricted * (−30%): 1: 150 g cod 3 times/week 2: 150 g cod 5 times/week 3: Control (no seafood)	Lean fish reduced WC Lean fish 5 t/week −3.4 Lean fish increased BP SBP: Lean fish 5 t/week +5 mmHg DBP: Lean fish 3 t/week +3 mmHg TG no association HDL no association
Telle-Hansen et al., 2012 [45] Norway	30 healthy adults (7 men and 23 women)	15 days, parallel 1: 150 g cod daily 2: 150 g salmon daily 3: Control (150 g potato daily)	Lean fish decreased TG −0.1 (*p* < 0.05) HDL no association Fatty fish decreased TG −0.2 (*p* < 0.05) Fatty fish increased HDL +0.1 (*p* < 0.05)
Thorsdottir et al., 2007 [46] Iceland, Spain, Ireland	324 overweight/obese healthy adults (138 men and 186 women)	8-week, parallel: Energy restricted * (−30%): 1: Lean fish (cod) 3 × 150 g/week 2: Fatty fish (salmon) 3 × 150 g/week3: Fish oil (DHA/EPA) 4: Control (no seafood)	Lean fish reduced WC Fatty fish reduced WC Lean fish −5.0 (2.9) (*p* < 0.05) Fatty fish −5.4 (3.3) (*p* < 0.05) Fish oil −5.1 (3.1) Control 4.0 (2.4)
Vazquez et al., 2014 [47] Spain	273 patients with metabolic syndrome	8-week, cross-over: 100 g/day lean fish (Namibia hake) Control: No fish/seafood	Lean fish reduced WC WC: treatment effect *p* < 0.001 Lean fish reduced DBP DBP: treatment effect *p* = 0.014 HDL no association TG no association

* −30% from calculated total energy expenditure, approximately 600 kcal/day. WC: Waist circumference, TG: Triglyceride, HDL-C: High-density lipoprotein cholesterol, BP: Blood pressure, SBP: Systolic blood pressure, DBP: Diastolic blood pressure, EPA: Eicosapentaenoic acid, DHA: Docosahexaenoic acid.

**Table 4:** Follow-up studies on fish consumption and the metabolic syndrome risk factors, assessing lean and fatty fish separately.

Reference/Country	Participants/Follow-Up Time		Results
Tørris et al., 2017 [48] Norway	23,907 adults from the Norwegian Tromsø Study, Tromsø 4 and 6, 13 years	Investigating fish consumption (≥1 ×/week compared to <1 ×/week) and change in MetS components by consumption of fish during the follow-up period, age adjusted	Lean fish decreased WC Men −1.15 (−1.96 to −0.35) Fatty fish increased WC Women 0.97 (0.29 to 1.65) Men 0.60 (0.01 to 1.18) Lean fish reduced TG Women −0.04 (−0.08 to −0.00) Men −0.11 (−0.17 to −0.06) Lean fish increased HDL Women 0.03 (0.01 to 0.05) Men 0.04 (0.02 to 0.05) Fatty fish increased HDL Men 0.02 (0.00 to 0.03) Lean fish decreased BP SBP Men −0.86 (−1.66 to −0.06) DBP Men −0.63 (−1.18 to −0.07)
Jakobsen et al., 2012 [49] Europe	89,432 adults from the European Prospective Investigation into Cancer and Nutrition (EPIC) study 5.5 years Investigating fish consumption and 1-year change in WC	1-year change in WC	Fatty fish decreased WC Annual WC change 0.01 cm/10 g higher fatty fish consumption per d (95% CI 0.02 to 0.01) Lean fish no association with WC

WC: Waist circumference, TG: Triglyceride, HDL-C: High-density lipoprotein cholesterol, BP: Blood pressure.

**Table 5:** Nutritional profile of commonly consumed, whole, raw fish, per 100 g [52].

Food Item, Raw	Energy	Fat	*n*-3	Taurine	Vitamin D	Selenium	Iodine
	kJ	g	g	mg	µg	µg	µg
Haddock (Melanogrammus aeglefinus)	290	0.2	0.05	28 ^b^/57 ^c^	0.5	30	320
Pollock (Pollachius pollachius)	279	0.2	0.05	-	2.2	30	143
Saithe (Pollachius virens)	292	0.3	0.1	162 ^c^	0.8	30	93
Cod (Gadus morhua)	343	1.1	0.26	108 ^a^/120 ^c^	2	22	119
Cod farmed (Gadus morhua)	358	0.5	0.16	-	0.7	30	300
Plaice (Pleuronectes platessa)	382	2.6	0.66	146 ^a^	6	30	14
Trout (Salmo trutta)	462	3.3	0.62	-	9	19	19
Mackerel May–June (Scomber scombrus)	516	5.4	1.38	-	6	30	50
Trout farmed (Salmo trutta)	693	10	2.47	-	6.9	30	5
Salmon wild (Salmo salar)	760	12	2.12	-	8	50	-
Salmon farmed (Salmo salar)	932	16	3.63	60 ^a^/94 ^c^	10	30	12
Mackerel, autumn (Scomber scombrus)	1214	25	6.35	78 ^a^	5.4	60	63

^a^ Taurine content (mg/100 g raw wet weight) a in portions of four fish species purchased in a supermarket [53]. ^b^ Taurine content (mg/100 g raw wet weight) in portions of 14 fish species (spot samples) [53]. ^c^ Taurine concentrations (mg/100 g wet sample) in unprocessed fresh samples [54].

**Table 6:** Current dietary recommendations from various governing bodies.

Organization/Diet	Dietary Fish Recommendation
Dietary Guidelines for Americans (2015–2020) [55]	Consumption of about 8 ounces (2–3 portions)/week of a variety of seafood (fish and shellfish), which provide an average consumption of 250 mg/day of EPA and DHA
Australian Dietary Guidelines 2013 [56]	At least two servings of fish/week
Dietary Guidelines for Chinese Residents (2016) [57]	The appropriate weekly intake is set at 280–525 g of fish
European Guidelines on cardiovascular disease prevention in clinical practice 2016 [8]	Fish 1–2 times/week, one of which to be fatty fish
Norwegian dietary recommendations (2014) [58]	At least 2–3 portions or 300–450 grams of fish weekly, including a minimum of 200 grams of fatty fish

8 ounces = 224 g (REF EFSA) [59]. EPA: eicosapentaenoic acid, DHA: docosahexaenoic acid.

## References

[1] Mendis S., Puska P., Norrving B. (2011). Global Atlas on Cardiovascular Disease Prevention and Control.

[2] World Health Organization (2011). Global Status Report on Noncommunicable Diseases 2014.

[3] Eckel R.H., Grundy S.M., Zimmet P.Z. (2005). The metabolic syndrome. Lancet.

[4] Alberti K.G., Eckel R.H., Grundy S.M., Zimmet P.Z., Cleeman J.I., Donato K.A., Fruchart J.-C., James W.P.T., Loria C.M., Smith S.C. (2009). Harmonizing the metabolic syndrome: A joint interim statement of the International Diabetes Federation Task Force on Epidemiology and Prevention; National Heart, Lung, and Blood Institute; American Heart Association; World Heart Federation; International Atherosclerosis Society; and International Association for the Study of Obesity. Circulation.

[5] Cameron A.J., Boyko E.J., Sicree R.A., Zimmet P.Z., Soderberg S., Alberti K.G., Tuomilehto J., Chitson P., Shaw J.E. (2008). Central obesity as a precursor to the metabolic syndrome in the AusDiab study and Mauritius. Obesity.

[6] Rask-Madsen C., Kahn C.R. (2012). Tissue-specific insulin signaling, metabolic syndrome, and cardiovascular disease. Arterioscler. Thromb. Vasc. Biol..

[7] Lea T. (2006). Immunologiog Immunologiske Teknikker.

[8] Piepoli M.F., Hoes A.W., Agewall S., Albus C., Brotons C., Catapano A.L., Cooney M.-T., Corra U., Cosyns B., Deaton C. (2016). 2016 European Guidelines on cardiovascular disease prevention in clinical practice. Eur. Heart J..

[9] Mozaffarian D., Appel L.J., Van Horn L. (2011). Components of a cardioprotective diet: New insights. Circulation.

[10] Skåre J.U., Brantsæter A.L., Frøyland L., Hemre G.-I., Knutsen H.K., Lillegaard I.T.L., Torstensen B. (2014). Benefit-Risk Assessment of Fish and Fish Products in the Norwegian Diet—An Update.

[11] Potenza M.V., Mechanick J.I. (2009). The metabolic syndrome: Definition, global impact, and pathophysiology. Nutr. Clin. Pract..

[12] Lund E.K. (2013). Health benefits of seafood; is it just the fatty acids?. Food Chem..

[13] Jayedi A., Shab-Bidar S., Eimeri S., Djafarian K. (2018). Fish consumption and risk of all-cause and cardiovascular mortality: A dose-response meta-analysis of prospective observational studies. Public Health Nutr..

[14] Raatz S.K., Silverstein J.T., Jahns L., Picklo M.J. (2013). Issues of fish consumption for cardiovascular disease risk reduction. Nutrients.

[15] Panagiotakos D.B., Zeimbekis A., Boutziouka V., Economou M., Kourlaba G., Toutouzas P., Polychronopoulos E. (2007). Long-term fish intake is associated with better lipid profile, arterial blood pressure, and blood glucose levels in elderly people from Mediterranean islands (MEDIS epidemiological study). Med. Sci. Monit..

[16] Strom M., Halldorsson T.I., Mortensen E.L., Torp-Pedersen C., Olsen S.F. (2012). Fish, *n*-3 fatty acids, and cardiovascular diseases in women of reproductive age: A prospective study in a large national cohort. Hypertension.

[17] Li Y.H., Zhou C.H., Pei H.J., Zhou X.L., Li L.H., Wu Y.J., Hui R.T. (2013). Fish consumption and incidence of heart failure: A meta-analysis of prospective cohort studies. Chin. Med. J..

[18] Chowdhury R., Stevens S., Gorman D., Pan A., Warnakula S., Chowdhury S., Ward H., Johnson L., Crowe F., Hu F.B. (2012). Association between fish consumption, long chain omega 3 fatty acids, and risk of cerebrovascular disease: Systematic review and meta-analysis. BMJ.

[19] Heine-Broring R.C., Brouwer I.A., Proenca R.V., van Rooij F.J., Hofman A., Oudkerk M., Witteman J.C.M., Geleijnse J.M. (2010). Intake of fish and marine *n*-3 fatty acids in relation to coronary calcification: The Rotterdam Study. Am. J. Clin. Nutr..

[20] Takata Y., Zhang X., Li H., Gao Y.T., Yang G., Gao J., Cai H., Xiang Y.-B., Zheng W., Shu X.-O. (2013). Fish intake and risks of total and cause-specific mortality in 2 population-based cohort studies of 134,296 men and women. Am. J. Epidemiol..

[21] Streppel M.T., Ocke M.C., Boshuizen H.C., Kok F.J., Kromhout D. (2008). Long-term fish consumption and *n*-3 fatty acid intake in relation to (sudden) coronary heart disease death: The Zutphen study. Eur. Heart J..

[22] Torris C., Molin M., Cvancarova Smastuen M. (2014). Fish consumption and its possible preventive role on the development and prevalence of metabolic syndrome—A systematic review. Diabetol. Metab. Syndr..

[23] Moher D., Liberati A., Tetzlaff J., Altman D.G., Group P. (2009). Preferred reporting items for systematic reviews and meta-analyses: The PRISMA statement. BMJ.

[24] Ramel A., Jonsdottir M.T., Thorsdottir I. (2009). Consumption of cod and weight loss in young overweight and obese adults on an energy reduced diet for 8-weeks. Nutr. Metab. Cardiovasc. Dis..

[25] Baik I., Abbott R.D., Curb J.D., Shin C. (2010). Intake of fish and *n*-3 fatty acids and future risk of metabolic syndrome. J. Am. Diet. Assoc..

[26] Kim Y.S., Xun P., Iribarren C., Van Horn L., Steffen L., Daviglus M.L., Siscovick D., Liu K., He K. (2016). Intake of fish and long-chain omega-3 polyunsaturated fatty acids and incidence of metabolic syndrome among American young adults: A 25-year follow-up study. Eur. J. Nutr..

[27] Karlsson T., Rosendahl-Riise H., Dierkes J., Drevon C.A., Tell G.S., Nygard O. (2017). Associations between fish intake and the metabolic syndrome and its components among middle-aged men and women: The Hordaland Health Study. Food Nutr. Res..

[28] Kouki R., Schwab U., Hassinen M., Komulainen P., Heikkila H., Lakka T.A., Rauramaa R. (2011). Food consumption, nutrient intake and the risk of having metabolic syndrome: The DR’s EXTRA Study. Eur. J. Clin. Nutr..

[29] Ruidavets J.B., Bongard V., Dallongeville J., Arveiler D., Ducimetiere P., Perret B., Simon C., Amouyel P., Ferrières J. (2007). High consumptions of grain, fish, dairy products and combinations of these are associated with a low prevalence of metabolic syndrome. J. Epidemiol. Community Health.

[30] Torris C., Molin M., Cvancarova M.S. (2016). Lean fish consumption is associated with lower risk of metabolic syndrome: A Norwegian cross sectional study. BMC Public Health.

[31] Torris C., Molin M., Cvancarova Smastuen M. (2016). Associations between fish consumption and metabolic syndrome. A large cross-sectional study from the Norwegian Tromso Study: Tromso 4. Diabetol. Metab. Syndr..

[32] Zaribaf F., Falahi E., Barak F., Heidari M., Keshteli A.H., Yazdannik A., Esmaillzadeh A. (2014). Fish consumption is inversely associated with the metabolic syndrome. Eur. J. Clin. Nutr..

[33] Kim Y.S., Xun P., He K. (2015). Fish consumption, long-chain omega-3 polyunsaturated fatty acid intake and risk of metabolic syndrome: A meta-analysis. Nutrients.

[34] Lai Y.H., Petrone A.B., Pankow J.S., Arnett D.K., North K.E., Ellison R.C., Hunt S.C., Djoussé L. (2013). Association of dietary omega-3 fatty acids with prevalence of metabolic syndrome: The National Heart, Lung, and Blood Institute Family Heart Study. Clin. Nutr..

[35] Pasalic D., Dodig S., Corovic N., Pizent A., Jurasovic J., Pavlovic M. (2011). High prevalence of metabolic syndrome in an elderly Croatian population—A multicentre study. Public Health Nutr..

[36] Aadland E.K., Lavigne C., Graff I.E., Eng O., Paquette M., Holthe A., Mellgren G., Jacques H., Liaset B. (2015). Lean-seafood intake reduces cardiovascular lipid risk factors in healthy subjects: Results from a randomized controlled trial with a crossover design. Am. J. Clin. Nutr..

[37] Bao D.Q., Mori T.A., Burke V., Puddey I.B., Beilin L.J. (1998). Effects of dietary fish and weight reduction on ambulatory blood pressure in overweight hypertensives. Hypertension.

[38] Erkkila A.T., Schwab U.S., de Mello V.D., Lappalainen T., Mussalo H., Lehto S., Kemi V., Lamberg-Allardt C., Uusitupa M.I.J. (2008). Effects of fatty and lean fish intake on blood pressure in subjects with coronary heart disease using multiple medications. Eur. J. Nutr..

[39] Hagen I.V., Helland A., Bratlie M., Brokstad K.A., Rosenlund G., Sveier H., Mellgren G., Gudbrandsen O.A. (2016). High intake of fatty fish, but not of lean fish, affects serum concentrations of TAG and HDL-cholesterol in healthy, normal-weight adults: A randomised trial. Br. J. Nutr..

[40] Hallund J., Madsen B.O., Bugel S.H., Jacobsen C., Jakobsen J., Krarup H., Holm J., Nielsen H.H., Lauritzen L. (2010). The effect of farmed trout on cardiovascular risk markers in healthy men. Br. J. Nutr..

[41] Lara J.J., Economou M., Wallace A.M., Rumley A., Lowe G., Slater C., Caslake M., Sattar N., Lean M.E.J. (2007). Benefits of salmon eating on traditional and novel vascular risk factors in young, non-obese healthy subjects. Atherosclerosis.

[42] Lindqvist H.M., Langkilde A.M., Undeland I., Sandberg A.S. (2009). Herring (*Clupea harengus*) intake influences lipoproteins but not inflammatory and oxidation markers in overweight men. Br. J. Nutr..

[43] Lindqvist H., Langkilde A.M., Undeland I., Radendal T., Sandberg A.S. (2007). Herring (*Clupea harengus*) supplemented diet influences risk factors for CVD in overweight subjects. Eur. J. Clin. Nutr..

[44] Ramel A., Martinez J.A., Kiely M., Bandarra N.M., Thorsdottir I. (2010). Moderate consumption of fatty fish reduces diastolic blood pressure in overweight and obese European young adults during energy restriction. Nutrition.

[45] Telle-Hansen V.H., Larsen L.N., Hostmark A.T., Molin M., Dahl L., Almendingen K., Ulven S.M. (2012). Daily intake of cod or salmon for 2 weeks decreases the 18:1n-9/18:0 ratio and serum triacylglycerols in healthy subjects. Lipids.

[46] Thorsdottir I., Tomasson H., Gunnarsdottir I., Gisladottir E., Kiely M., Parra M.D., Bandarra N.M., Schaafsma G., Martinéz J.A. (2007). Randomized trial of weight-loss-diets for young adults varying in fish and fish oil content. Int. J. Obes..

[47] Vazquez C., Botella-Carretero J.I., Corella D., Fiol M., Lage M., Lurbe E., Richart C., Fernández-Real J.M., Fuentes F., Ordóñez A. (2014). White fish reduces cardiovascular risk factors in patients with metabolic syndrome: The WISH-CARE study, a multicenter randomized clinical trial. Nutr. Metab. Cardiovasc. Dis..

[48] Torris C., Molin M., Smastuen M.C. (2017). Lean Fish Consumption Is Associated with Beneficial Changes in the Metabolic Syndrome Components: A 13-Year Follow-Up Study from the Norwegian Tromso Study. Nutrients.

[49] Jakobsen M.U., Due K.M., Dethlefsen C., Halkjaer J., Holst C., Forouhi N.G., Tjønneland A., Boeing H., Buijsse B., Palli D. (2012). Fish consumption does not prevent increase in waist circumference in European women and men. Br. J. Nutr..

[50] Lankinen M., Schwab U., Kolehmainen M., Paananen J., Poutanen K., Mykkanen H., Seppänen-Laakso T., Gylling H., Uusitupa M., Orešič M. (2011). Whole grain products, fish and bilberries alter glucose and lipid metabolism in a randomized, controlled trial: The Sysdimet study. PLoS ONE.

[51] Uusitupa M., Hermansen K., Savolainen M.J., Schwab U., Kolehmainen M., Brader L., Mortensen L.S., Cloetens L., Johansson-Persson A., Önning G. (2013). Effects of an isocaloric healthy Nordic diet on insulin sensitivity, lipid profile and inflammation markers in metabolic syndrome—A randomized study (SYSDIET). J. Intern. Med..

[52] Norwegian Food Safety Authority, The Norwegian Directorate of Health, University of Oslo Norwegian Food Composition Database 2016. www.matvaretabellen.no.

[53] Gormley T., Neumann T., Fagan J., Brunton N. (2007). Taurine content of raw and processed fish fillets/portions. Eur. Food Res. Technol..

[54] Dragnes B.T., Larsen R., Ernstsen M.H., Maehre H., Elvevoll E.O. (2009). Impact of processing on the taurine content in processed seafood and their corresponding unprocessed raw materials. Int. J. Food Sci. Nutr..

[55] U.S. Department of Health and Human Services, U.S. Department of Agriculture (2015). 2015–2020 Dietary Guidelines for Americans.

[56] Brownie S., Muggleston H., Oliver C. (2015). The 2013 Australian dietary guidelines and recommendations for older Australians. Aust. Fam. Phys..

[57] Wang S., Lay S., Yu H., Shen S. (2016). Dietary Guidelines for Chinese Residents (2016): Comments and comparisons. Biomed. Biotechnol..

[58] The Norwegian Directorate of Health (2014). Anbefalinger om Kosthold, Ernæring og Fysisk Aktivitet.

[59] European Food Safety Authority (EFSA) (2017). Dietary Reference Values for Nutrients. https://www.efsa.europa.eu/sites/default/files/2017_09_DRVs_summary_report.pdf.

[60] Uhe A.M., Collier G.R., O’Dea K. (1992). A comparison of the effects of beef, chicken and fish protein on satiety and amino acid profiles in lean male subjects. J. Nutr..

[61] Borzoei S., Neovius M., Barkeling B., Teixeira-Pinto A., Rossner S. (2006). A comparison of effects of fish and beef protein on satiety in normal weight men. Eur. J. Clin. Nutr..

[62] Pal S., Ellis V. (2010). The acute effects of four protein meals on insulin, glucose, appetite and energy intake in lean men. Br. J. Nutr..

[63] Kritchevsky D., Tepper S.A., Czarnecki S.K., Klurfeld D.M. (1982). Atherogenicity of animal and vegetable protein: Influence of the lysine to arginine ratio. Atherosclerosis.

[64] Hosomi R., Fukunaga K., Arai H., Kanda S., Nishiyama T., Yoshida M. (2012). Fish protein hydrolysates affect cholesterol metabolism in rats fed non-cholesterol and high-cholesterol diets. J. Med. Food.

[65] Wergedahl H., Liaset B., Gudbrandsen O., Lied E. (2004). Fish Protein Hydrolysate Reduces Plasma Total Cholesterol, Increases the Proportion of HDL Cholesterol, and Lowers Acyl-CoA: Cholesterol Acyltransferase Activity in Liver of Zucker Rats1. J. Nutr..

[66] Drotningsvik A., Mjos S.A., Pampanin D.M., Slizyte R., Carvajal A., Remman T., Høgøy I., Gudbrandsen O.A. (2016). Dietary fish protein hydrolysates containing bioactive motifs affect serum and adipose tissue fatty acid compositions, serum lipids, postprandial glucose regulation and growth in obese Zucker fa/fa rats. Br. J. Nutr..

[67] El Khoury D., Anderson G.H. (2013). Recent advances in dietary proteins and lipid metabolism. Curr. Opin. Lipidol..

[68] Ouellet V., Marois J., Weisnagel S.J., Jacques H. (2007). Dietary cod protein improves insulin sensitivity in insulin-resistant men and women: A randomized controlled trial. Diabetes Care.

[69] Yamori Y., Taguchi T., Hamada A., Kunimasa K., Mori H., Mori M. (2010). Taurine in health and diseases: Consistent evidence from experimental and epidemiological studies. J. Biomed. Sci..

[70] Imae M., Asano T., Murakami S. (2014). Potential role of taurine in the prevention of diabetes and metabolic syndrome. Amino Acids.

[71] Murakami S. (2014). Taurine and atherosclerosis. Amino Acids.

[72] Yamori Y., Taguchi T., Mori H., Mori M. (2010). Low cardiovascular risks in the middle aged males and females excreting greater 24-hour urinary taurine and magnesium in 41 WHO-CARDIAC study populations in the world. J. Biomed. Sci..

[73] Zhang M., Bi L.F., Fang J.H., Su X.L., Da G.L., Kuwamori T., Kuwamori T., Kagamimori S. (2004). Beneficial effects of taurine on serum lipids in overweight or obese non-diabetic subjects. Amino Acids.

[74] Elvevoll E.O., Eilertsen K.E., Brox J., Dragnes B.T., Falkenberg P., Olsen J.O., Kirkhus B., Lamglait A., Østerud B. (2008). Seafood diets: Hypolipidemic and antiatherogenic effects of taurine and *n*-3 fatty acids. Atherosclerosis.

[75] Murakami S. (2017). The physiological and pathophysiological roles of taurine in adipose tissue in relation to obesity. Life Sci..

[76] Rosa F., Freitas E., Deminice R., Jordão A., Marchini J. (2014). Oxidative stress and inflammation in obesity after taurine supplementation: A double-blind, placebo-controlled study. Eur. J. Nutr..

[77] Sun Q., Wang B., Li Y., Sun F., Li P., Xia W., Zhou X., Li Q., Wang X., Chen J. (2016). Taurine Supplementation Lowers Blood Pressure and Improves Vascular Function in Prehypertension: Randomized, Double-Blind, Placebo-Controlled Study. Hypertension.

[78] Maia A.R., Batista T.M., Victorio J.A., Clerici S.P., Delbin M.A., Carneiro E.M., Davel A.P. (2014). Taurine supplementation reduces blood pressure and prevents endothelial dysfunction and oxidative stress in post-weaning protein-restricted rats. PLoS ONE.

[79] Xu Y.J., Arneja A.S., Tappia P.S., Dhalla N.S. (2008). The potential health benefits of taurine in cardiovascular disease. Exp. Clin. Cardiol..

[80] Ito T., Yoshikawa N., Ito H., Schaffer S.W. (2015). Impact of taurine depletion on glucose control and insulin secretion in mice. J. Pharmacol. Sci..

[81] Kris-Etherton P.M., Fleming J.A. (2015). Emerging nutrition science on fatty acids and cardiovascular disease: Nutritionists’ perspectives. Adv. Nutr..

[82] Wiktorowska-Owczarek A., Berezinska M., Nowak J.Z. (2015). PUFAs: Structures, Metabolism and Functions. Adv. Clin. Exp. Med..

[83] Back M. (2017). Omega-3 fatty acids in atherosclerosis and coronary artery disease. Future Sci. OA.

[84] Martinez-Fernandez L., Laiglesia L.M., Huerta A.E., Martinez J.A., Moreno-Aliaga M.J. (2015). Omega-3 fatty acids and adipose tissue function in obesity and metabolic syndrome. Prostaglandins Other Lipid Mediat..

[85] Bang H.O. (1990). Lipid Research in Greenland. Preventive and Therapeutic Consequences. Scand. J. Public Health.

[86] Mori T.A. (2017). Marine OMEGA-3 fatty acids in the prevention of cardiovascular disease. Fitoterapia.

[87] Dunn S.L., Siu W., Freund J., Boutcher S.H. (2014). The effect of a lifestyle intervention on metabolic health in young women. Diabetes Metab. Syndr. Obes. Targets Ther..

[88] Lee T.C., Ivester P., Hester A.G., Sergeant S., Case L.D., Morgan T., Kouba E.O., Chilton F.H. (2014). The impact of polyunsaturated fatty acid-based dietary supplements on disease biomarkers in a metabolic syndrome/diabetes population. Lipids Health Dis..

[89] Pedersen M.H., Molgaard C., Hellgren L.I., Lauritzen L. (2010). Effects of fish oil supplementation on markers of the metabolic syndrome. J. Pediatr..

[90] Aung T., Halsey J., Kromhout D., Gerstein H.C., Marchioli R., Tavazzi L., Geleijnse J.M., Rauch B., Ness A., Galan P. (2018). Associations of Omega-3 Fatty Acid Supplement Use With Cardiovascular Disease Risks: Meta-analysis of 10 Trials Involving 77 917 Individuals. JAMA Cardiol..

[91] Tortosa-Caparros E., Navas-Carrillo D., Marin F., Orenes-Pinero E. (2017). Anti-inflammatory effects of omega 3 and omega 6 polyunsaturated fatty acids in cardiovascular disease and metabolic syndrome. Crit. Rev. Food Sci. Nutr..

[92] Mullen A., Loscher C.E., Roche H.M. (2010). Anti-inflammatory effects of EPA and DHA are dependent upon time and dose-response elements associated with LPS stimulation in THP-1-derived macrophages. J. Nutr. Biochem..

[93] Romacho T., Glosse P., Richter I., Elsen M., Schoemaker M.H., van Tol E.A., Eckel J. (2015). Nutritional ingredients modulate adipokine secretion and inflammation in human primary adipocytes. Nutrients.

[94] Saravanan P., Davidson N.C., Schmidt E.B., Calder P.C. (2010). Cardiovascular effects of marine omega-3 fatty acids. Lancet.

[95] Visioli F., Rise P., Barassi M.C., Marangoni F., Galli C. (2003). Dietary intake of fish vs. formulations leads to higher plasma concentrations of *n*-3 fatty acids. Lipids.

[96] Ebbesson S.O., Tejero M.E., Nobmann E.D., Lopez-Alvarenga J.C., Ebbesson L., Romenesko T., Carter E.A., Resnick H.E., Devereux R.B., MacCluer J.W. (2007). Fatty acid consumption and metabolic syndrome components: The GOCADAN study. J. Cardiometab. Syndr..

[97] Hoe E., Nathanielsz J., Toh Z.Q., Spry L., Marimla R., Balloch A., Mulholland K., Licciardi P.V. (2016). Anti-Inflammatory Effects of Vitamin D on Human Immune Cells in the Context of Bacterial Infection. Nutrients.

[98] Lehmann U., Gjessing H.R., Hirche F., Mueller-Belecke A., Gudbrandsen O.A., Ueland P.M., Mellgren G., Lauritzen L., Lindqvist H., Hansen A.L. (2015). Efficacy of fish intake on vitamin D status: A meta-analysis of randomized controlled trials. Am. J. Clin. Nutr..

[99] De Roos B., Sneddon A.A., Sprague M., Horgan G.W., Brouwer I.A. (2017). The potential impact of compositional changes in farmed fish on its health-giving properties: Is it time to reconsider current dietary recommendations?. Public Health Nutr..

[100] Cashman K.D. (2015). Vitamin D: Dietary requirements and food fortification as a means of helping achieve adequate vitamin D status. J. Steroid Biochem. Mol. Biol..

[101] Cashman K.D., Dowling K.G., Skrabakova Z., Gonzalez-Gross M., Valtuena J., De Henauw S., Moreno L., Damsgaard C.T., Michaelsen K.F., Mølgaard C. (2016). Vitamin D deficiency in Europe: Pandemic?. Am. J. Clin. Nutr..

[102] Conzade R., Koenig W., Heier M., Schneider A., Grill E., Peters A., Thorand B. (2017). Prevalence and Predictors of Subclinical Micronutrient Deficiency in German Older Adults: Results from the Population-Based KORA-Age Study. Nutrients.

[103] Forrest K.Y., Stuhldreher W.L. (2011). Prevalence and correlates of vitamin D deficiency in US adults. Nutr. Res..

[104] Schmitt E.B., Nahas-Neto J., Bueloni-Dias F., Poloni P.F., Orsatti C.L., Petri Nahas E.A. (2018). Vitamin D deficiency is associated with metabolic syndrome in postmenopausal women. Maturitas.

[105] Pan G.T., Guo J.F., Mei S.L., Zhang M.X., Hu Z.Y., Zhong C.K., Zeng C.Y., Liu X.H., Ma Q.H., Li B.Y. (2016). Vitamin D Deficiency in Relation to the Risk of Metabolic Syndrome in Middle-Aged and Elderly Patients with Type 2 Diabetes Mellitus. J. Nutr. Sci. Vitaminol..

[106] Elizondo-Montemayor L., Castillo E.C., Rodriguez-Lopez C., Villarreal-Calderon J.R., Gomez-Carmona M., Tenorio-Martinez S., Nieblas B., García-Rivas G. (2017). Seasonal Variation in Vitamin D in Association with Age, Inflammatory Cytokines, Anthropometric Parameters, and Lifestyle Factors in Older Adults. Mediat. Inflamm..

[107] Ganji V., Zhang X., Shaikh N., Tangpricha V. (2011). Serum 25-hydroxyvitamin D concentrations are associated with prevalence of metabolic syndrome and various cardiometabolic risk factors in US children and adolescents based on assay-adjusted serum 25-hydroxyvitamin D data from NHANES 2001–2006. Am. J. Clin. Nutr..

[108] Giovinazzo S., Alibrandi A., Campenni A., Trimarchi F., Ruggeri R.M. (2017). Correlation of cardio-metabolic parameters with vitamin D status in healthy premenopausal women. J. Endocrinol. Investig..

[109] Kang J.Y., Kim M.K., Jung S., Shin J., Choi B.Y. (2016). The cross-sectional relationships of dietary and serum vitamin D with cardiometabolic risk factors: Metabolic components, subclinical atherosclerosis, and arterial stiffness. Nutrition.

[110] Kim Y.S., Hwang J.H., Song M.R. (2018). The Association Between Vitamin D Deficiency and Metabolic Syndrome in Korean Adolescents. J. Pediatr. Nurs..

[111] Schomburg L., Köhrle J., Diamond A.M. (2008). On the importance of selenium and iodine metabolism for thyroid hormone biosynthesis and human health. Mol. Nutr. Food Res..

[112] World Health Organization, Food and Agriculture Organization of the United Nations (2004). Vitamin & Mineral Requirements in Human Nutrition.

[113] Reinehr T. (2010). Obesity and thyroid function. Mol. Cell. Endocrinol..

[114] Brough L., Gunn C., Weber J., Coad J., Jin Y., Thomson J., Mauze M., Kruger M.C. (2017). Iodine and Selenium Intakes of Postmenopausal Women in New Zealand. Nutrients.

[115] Nystrom H.F., Brantsaeter A.L., Erlund I., Gunnarsdottir I., Hulthen L., Laurberg P., Mattisson I., Rasmussen L.B., Virtanen S., Meltzer H.M. (2016). Iodine status in the Nordic countries—Past and present. Food Nutr Res..

[116] Henjum S., Lilleengen A.M., Aakre I., Dudareva A., Gjengedal E.L.F., Meltzer H.M., Brantsæter A.L. (2017). Suboptimal Iodine Concentration in Breastmilk and Inadequate Iodine Intake among Lactating Women in Norway. Nutrients.

[117] Kirkegaard-Klitbo D.M., Perslev K., Andersen S.L., Perrild H., Knudsen N., Weber T., Rasmussen L.B., Laurberg P. (2016). Iodine deficiency in pregnancy is prevalent in vulnerable groups in Denmark. Dan. Med. J..

[118] Tam W.H., Chan R.S., Chan M.H., Yuen L.Y., Li L., Sea M.M., Woo J. (2017). Moderate iodine deficiency among pregnant women in Hong Kong: Revisit the problem after two decades. Hong Kong Med. J..

[119] Gunnarsdottir I., Gustavsdottir A.G., Steingrimsdottir L., Maage A., Johannesson A.J., Thorsdottir I. (2013). Iodine status of pregnant women in a population changing from high to lower fish and milk consumption. Public Health Nutr..

[120] Ayturk S., Gursoy A., Kut A., Anil C., Nar A., Tutuncu N.B. (2009). Metabolic syndrome and its components are associated with increased thyroid volume and nodule prevalence in a mild-to-moderate iodine-deficient area. Eur. J. Endocrinol..

[121] Lecube A., Zafon C., Gromaz A., Fort J., Caubet E., Baena J., Tortosa F. (2015). Iodine Deficiency Is Higher in Morbid Obesity in Comparison with Late After Bariatric Surgery and Non-obese Women. Obes. Surg..

[122] Stoffaneller R., Morse N.L. (2015). A review of dietary selenium intake and selenium status in Europe and the Middle East. Nutrients.

[123] Alfthan G., Eurola M., Ekholm P., Venalainen E.R., Root T., Korkalainen K., Hartikainen H., Salminen P., Hietaniemi V., Aspila P. (2015). Effects of nationwide addition of selenium to fertilizers on foods, and animal and human health in Finland: From deficiency to optimal selenium status of the population. J. Trace Elements Med. Biol..

[124] Olza J., Aranceta-Bartrina J., Gonzalez-Gross M., Ortega R.M., Serra-Majem L., Varela-Moreiras G., Gil Á. (2017). Reported Dietary Intake and Food Sources of Zinc, Selenium, and Vitamins A, E and C in the Spanish Population: Findings from the ANIBES Study. Nutrients.

[125] Nawrot T.S., Staessen J.A., Roels H.A., Den Hond E., Thijs L., Fagard R.H., Struijker-Boudier H.A. (2007). Blood pressure and blood selenium: A cross-sectional and longitudinal population study. Eur. Heart J..

[126] Park K., Seo E. (2016). Association between Toenail Mercury and Metabolic Syndrome Is Modified by Selenium. Nutrients.

